# Increased mitochondrial biogenesis preserves intestinal stem cell homeostasis and contributes to longevity in *Indy* mutant flies

**DOI:** 10.18632/aging.100658

**Published:** 2014-05-06

**Authors:** Ryan P. Rogers, Blanka Rogina

**Affiliations:** Department of Genetics and Developmental Biology, School of Medicine, University of Connecticut Health Center, 263 Farmington, CT 06030-6403, USA

**Keywords:** Indy, intestinal stem cells, caloric restriction, Drosophila, aging, mitochondria

## Abstract

The *Drosophila Indy (I'm Not Dead Yet)* gene encodes a plasma membrane transporter of Krebs cycle intermediates, with robust expression in tissues associated with metabolism. Reduced INDY alters metabolism and extends longevity in a manner similar to caloric restriction (CR); however, little is known about the tissue specific physiological effects of INDY reduction. Here we focused on the effects of INDY reduction in the *Drosophila* midgut due to the importance of intestinal tissue homeostasis in healthy aging and longevity. The expression of *Indy* mRNA in the midgut changes in response to aging and nutrition. Genetic reduction of *Indy* expression increases midgut expression of the mitochondrial regulator *spargel/dPGC*-1, which is accompanied by increased mitochondrial biogenesis and reduced reactive oxygen species (ROS). These physiological changes in the *Indy* mutant midgut preserve intestinal stem cell (ISC) homeostasis and are associated with healthy aging. Genetic studies confirm that *dPGC-1* mediates the regulatory effects of INDY, as illustrated by lack of longevity extension and ISC homeostasis in flies with mutations in both *Indy* and *dPGC1*. Our data suggest INDY may be a physiological regulator that modulates intermediary metabolism in response to changes in nutrient availability and organismal needs by modulating *dPGC-1*

## INTRODUCTION

Caloric restriction (CR) extends lifespan in nearly all species and promotes organismal energy balance by affecting intermediary metabolism and mitochondrial biogenesis [1-4]. Interventions that alter intermediary metabolism are though to extend longevity by preserving the balance between energy production and free radical production [1, 5, 6]. *Indy* (*I'm Not Dead, Yet*) encodes a plasma membrane protein that transports Krebs' cycle intermediates across tissues associated with intermediary metabolism [7-10]. Reduced *Indy*–mediated transport extend longevity in worms and flies by decreasing the uptake and utilization of nutrients and altering intermediate nutrient metabolism in a manner similar to CR [6, 8, 10-14]. Furthermore, it was shown that caloric content of food directly affects *Indy* expression in fly heads and thoraces, suggesting a direct relationship between INDY and metabolism [14].

*dPGC-1/spargel* is the *Drosophila* homolog of mammalian PGC-1, a transcriptional co-activator that promotes mitochondrial biogenesis by increasing the expression of genes encoding mitochondrial proteins [15, 16]. Upregulation of *dPGC-1* is a hallmark of CR-mediated longevity and is thought to represent a response mechanism to compensate for energetic deficits caused by limited nutrient availability [2, 16]. Increases in *dPGC-1* preserve mitochondrial functional efficiency without consequential changes in ROS. Previous analyses of *Indy* mutant flies revealed upregulation of mitochondrial biogenesis mediated by increased levels of *dPGC-1* in heads and thoraces [6].

Recently, *dPGC-1* upregulation in stem and progenitor cells of the digestive tract was shown to preserve intestinal stem cell (ISC) proliferative homeostasis and extend lifespan [17]. The *Drosophila* midgut is regenerated by multipotent ISCs, which replace damaged epithelial tissue in response to injury, infection or changes in redox environment [18-22]. Low levels of reactive oxygen species (ROS) maintain stemness, self-renewal and multipotency in ISCs; whereas, age-associated ROS accumulation induces continuous activation marked by ISC hyper-proliferation and loss of intestinal integrity [18].

Here we describe a role for *Indy* as a physiological regulator that modulates expression in response to changes in nutrient availability. This is illustrated by altered *Indy* expression in flies following changes in caloric content and at later ages suggesting that INDY-mediated transport is adjusted in an effort to meet energetic demands. Further, we characterized role for *dPGC-1* in mediating the downstream regulatory effects of INDY reduction, such as the observed changes in *Indy* mutant mitochondrial physiology, oxidative stress resistance and reduction of ROS levels. Longevity studies support a role for *dPGC-1* as a downstream effector of *Indy* mutations as shown by overlapping longevity pathways and absence of lifespan extension without wild-type levels of *dPGC-1*. Our findings show that *Indy* mutations affect intermediary metabolism to preserve energy balance in response to altered nutrient availability, which by affecting the redox environment of the midgut promotes healthy aging.

## RESULTS

### Aging increases *Indy* mRNA levels in the midgut of control flies

**Figure 1:**
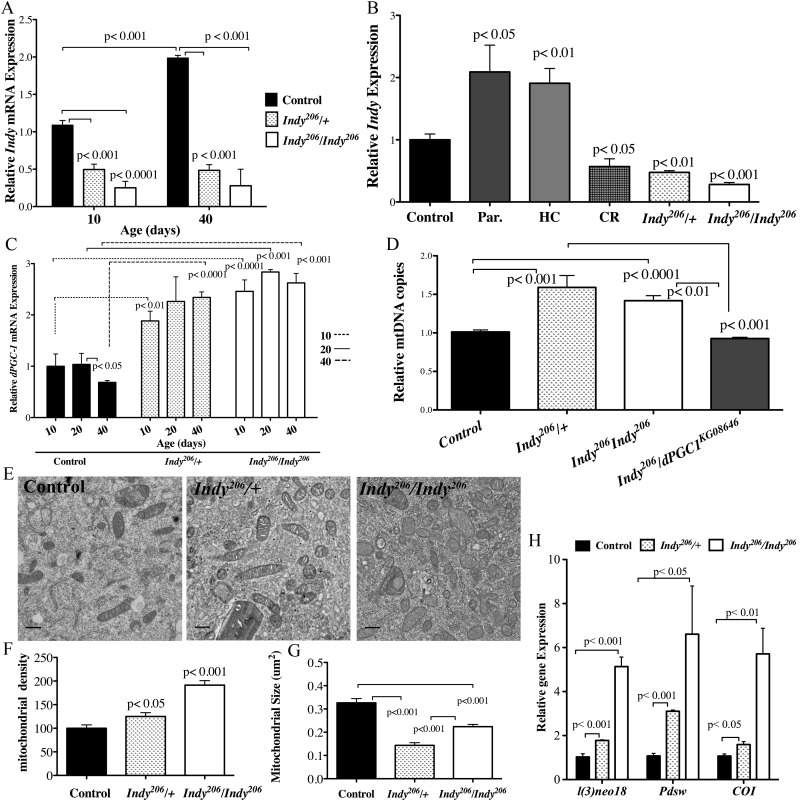
*Indy* reduction is associated with increased *dPGC-1* levels and mitochondrial biogenesis. (**A**) *Indy* mRNA levels in the midgut of *yw* control, *Indy^206^/+* and *Indy^206^/Indy^206^* female flies at 10 and 40 days determined by qPCR. Controls show an age-related increase in *Indy* mRNA, which is absent in the mutant midgut (n=3, 25 guts per replicate. p<0.001, p<0.0001, t test, error bars represent SEM). (**B**) *Indy* mRNA levels in *yw* control flies on a regular diet, after overnight exposure to paraquat, HC and CR, and *Indy^206^/+* and *Indy^206^/Indy^206^* mutant flies at 20 days. Paraquat and HC significantly increases and CR significantly reduces *Indy* transcript levels in the midgut of control female flies (n=3, 25 guts per replicate p<0.01, p<0.05, p<0.001, error bars represent SEM). (**C**) *dPGC-1* mRNA levels in female *yw* control, *Indy^206^/+* and *Indy^206^/Indy^206^* mutant midguts at 10, 20 and 40 days. There is a significant age-related decrease in dPGC-1 mRNA in control flies by 40 days (p <0.05, t test) that is absent in heterozygous and homozygous *Indy^206^* mutants, which show increased dPGC-1 mRNA levels compared to controls at all ages (p<0.01, p<0.001, p<0.0001, t test. n=3, 25 guts per replicate, error bars represent SEM). (**D**) Mitochondrial (COI) and nuclear (GAPDH) DNA ratio determined by qPCR. *Indy^206^/+* and *Indy^206^/Indy^206^* mutant females show significant increases in mitochondrial DNA copy number, compared to yw control or *Indy^206^/dPGC-1^KG08646^* mutant flies (p<0.01, p<0.001, p<0.0001, n=3, 25 guts per replicate) (**E**) Electron micrographs of control, *Indy^206^/+* and *Indy^206^/Indy^206^* midguts at 20 days imaged at 10,000x. Scale bar represents 11μm. (**F**) Mitochondrial density (mitochondrial number/counted cell volume X100) as assessed by point counting in Image J. There is a significant increase in mitochondrial number in *Indy^206^/+* and *Indy^206^/Indy^206^* mutant midgut tissue at 20 days (p<0.05, p<0.001, t test. n> 25 cells per guts). (**G**) *Indy^206^/+* and *Indy^206^/Indy^206^* mutants have significantly smaller mitochondria at 20 days in midgut tissue assessed by point counting in Image J. (p<0.001, n>25 cells per gut). (**H**) Quantification of *l(2)neo, Pdsw* encoding components of complex I and *Cytochrome C oxidase* encoding a component of complex IV are increased in the midgut of *Indy^206^/+* and *Indy^206^/Indy^206^* mutant flies determined by qPCR. (p<0.05, p<0.01, p<0.001, n=3, 25 guts per replicate).

In *Drosophila*, INDY is predominantly expressed in the basolateral membrane of the midgut epithelia, fat body and oenocytes [10]. To identify a relationship between *Indy* expression and aging in midgut tissue, we measured *Indy* mRNA levels in control *yellow-white* (*yw*) flies at 10 and 40 days. *Indy* transcript levels increase by approximately 89% in female *yw* flies, whereas *Indy^206^/+* and *Indy^206^/Indy^206^* mutants show decreased *Indy* mRNA levels at all ages following 10 generations of backcrossing into *yw* background (Fig. 1A, S1A). Consistently, exposure to 20 mM paraquat, an agent known to induce free radical production and mimic aging, upregulates *Indy* mRNA and protein levels in young control flies to levels similar to those that are observed in aged flies (Fig. 1B, S1B, S1C) [5, 19].

To investigate the relationship between *Indy* mRNA and nutrient availability in the midgut, we measured *Indy* mRNA levels in female *yw* control, *Indy^206^/+* and *Indy^206^/Indy^206^* flies on a normal, high caloric (HC) and CR diets at 20 days. *Indy* mRNA levels nearly doubled in *yw* flies on a HC diet, whereas a 50% reduction in transcript was observed in both *yw* flies on a CR diet and *Indy^206^/+* flies on a regular diet (Fig. 1B). Diet-induced changes in *Indy* mRNA levels support a role for *Indy* as a physiological regulator, whose expression changes to modulate intermediate metabolism in response to nutrient availability.

### *Indy* reduction is associated with increased *dPGC-1* levels in the midgut

*dPGC-1* increases mitochondrial biogenesis in response to CR and decreases as a consequnce of normal aging [2, 3, 6, 7, 16]. Consistently, we found a significant age-related decrease in *dPGC-1* mRNA levels in the midgut of *yw* females between 20 and 40 days (Fig. 1C). Our observation that *Indy* levels decrease in response to CR (Fig. 1B), led us to investigate whether INDY reduction is sufficient to upregulate *dPGC-1* in the fly midgut and rescue the age-associated decline in expression levels. We found significantly higher *dPGC-*1 mRNA levels compared *yw* controls at all ages in both *Indy^206^/+* and *Indy^206^/Indy^206^* mutant females, with a similar increase observed in *Indy^206^/+* male flies at age 40 (Fig. 1C, S2A). This trend is consistent through out lifespan, as demonstrated by the absence of any age-associated changes in both *Indy* and *dPGC-1* mRNA levels in *Indy* mutant midguts (Fig. 1A, 1C).

To further examine the relationship between *Indy* and *dPGC-1* mRNA levels, we used the *TIGS2*-geneswitch driver (*TIGS2-GS*) to drive gut specific *Indy-*RNAi mediated silencing. *dPGC-1* mRNA levels increase in response to a small reduction of *Indy* mRNA levels in the midguts of *TIGS2-GS; Indy-*RNAi male and female flies at 20 days (Fig. S2B-E). Together, these data suggest that there is an inverse relationship between *Indy* and *dPGC-1* mRNA levels in the midgut.

### Reduced *Indy* increases *dPGC-1* medited mitochondrial biogenesis

We next examined whether *dPGC-1* upregulation in the midgut of *Indy* mutant flies was sufficient to increase mitochondrial biogenesis by measuring mitochondrial density. The ratio of mitochondrial DNA to nuclear DNA in the midgut of *Indy^206^/+* and *Indy^206^/Indy^206^* mutant flies is significantly increased compared to control at 40 days (Fig. 1D). Using double mutant flies with the *Indy^206^* mutant allele and a hypomorphic *dPGC-1* allele *(Indy^206^/dPGC-1^KG08646^)*, we determined that the observed increase in mitochondrial DNA copy number, which depends on increased *dPGC-1* levels. These flies have mitochondrial DNA copy numbers similar to those observed in control flies at 40 days (Fig. 1D). Increased mitochondrial biogenesis in enterocytes residing in the anterior midgut of *Indy* mutant was confirmed by electron microscopy and the point counting method. Enterocytes comprise ~90% of the midgut cell populations; therefore thery represent the majority of cell types and can be used to indicate overal mitochondrial density in the midgut. Electron micrographs of *Indy^206^/+* and *Indy^206^/Indy^206^* mutant midgut tissue show a clear increase in mitochondrial density and significantly smaller size by 20 days compared to controls (Fig. 1E-G).

In addition to higher mitochondrial biogenesis, *Indy^206^/+* and *Indy^206^/Indy^206^* female midguts have increases in mitochondrial electron transport chain complex I (ETC) gene expression (Figure 1H). While *l(3)neo18* and *Pdsw* mRNA levels are elevated in both *Indy^206^/+* and *Indy^206^/Indy^206^*, we also observed significant increases in *ND23, ND42*, *ND75* mRNA levels in the midgut of *Indy^206^/Indy^206^* mutant flies by 20 days (Fig. S2F). Furthermore, mRNA levels of ETC complex IV component, *Cytochrome C oxidase I (COI)*, were also significantly increased in aged *Indy^206^/+* and *Indy^206^/Indy^206^* female flies (Fig. 1H).

### *Indy* mutants have enhanced mitochondrial activity and reduced ROS levels in the midgut

**Figure 2:**
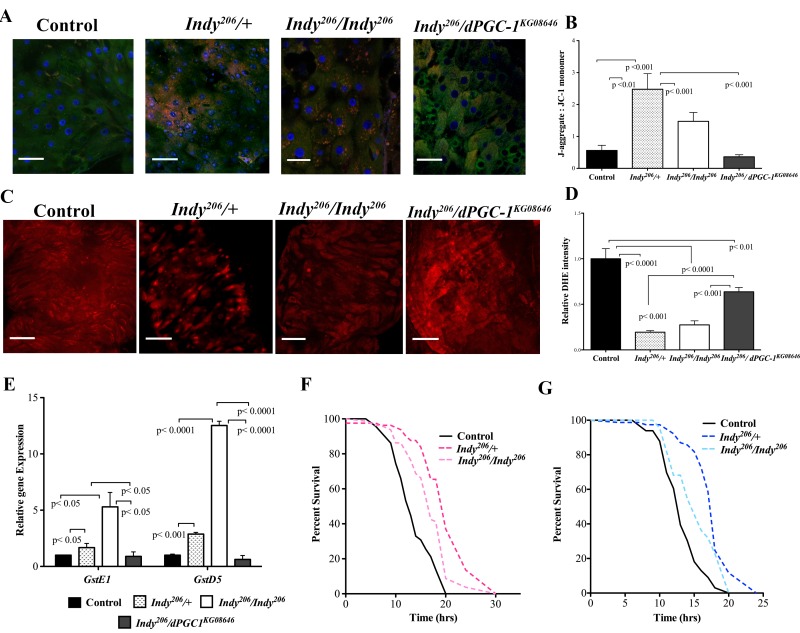
*Indy* mutants have reduced ROS levels and increased oxidative stress resistance. (**A**) Visualization of JC-1 dye in female *yw* control, *Indy^206^/+*, *Indy^206^/Indy^206^* and *Indy^206^*/*dPGC-1^KG08646^* mutant flies at 40 days. *Indy* mutant flies show increased mitochondrial membrane potential compared to controls and *Indy^206^*/*dPGC-1^KG08646^* mutants. Scale bar represents 1μm (**B**) Ratio of red JC-aggregates to green JC-1 monomer. *Indy^206^/+* and *Indy^206^/Indy^206^* mutant flies have increased mitochondrial membrane potential shown by increased red:green JC-1 ratiocompared to control and *Indy^206^*/*dPGC-1^KG08646^* mutants (p<0.01, p<0.001, n>10 guts per genotype, compared by Mann-Whitney U test) (**C**) Positive DHE staining for ROS in compressed Z-stack of female control, *Indy^206^/+*, *Indy^206^/Indy^206^* and *Indy^206^*/*dPGC-1^KG08646^* mutant midgut flies at 40 days. Scale bar represents 1μm. (**D**) Mean DHE intensity in compressed Z-stack of female control, *Indy^206^/+, Indy^206^/Indy^206^* and *Indy^206^*/*dPGC-1^KG08646^* mutant midguts at 40 days. (p<0.001, p<0.0001, n>15 guts per genotype; Scale bar represents 1μm). (**E**) Levels of *GstE1* and *GstD5* mRNA in female *yw* control, *Indy^206^/+*, *Indy^206^/Indy^206^* and *Indy^206^*/*dPGC-1^KG08646^* mutant midgut tissue determined by qPCR. There is a significant increase (p<0.05, p<0.001, p< 0001 n=3, 25 guts per replicate) in levels of both gene mRNA levels in *Indy^206^* mutant midgut tissue at 20 days compared to control and *Indy^206^*/*dPGC-1^KG08646^*. Survival curves for female (**F**) and male (**G**) *yw* control, *Indy^206^/+* and *Indy^206^/Indy^206^* flies on 20mM paraquat. *Indy^206^* mutants have increased resistance to oxidative stress compared to control.

High mitochondrial membrane potential is associated with preserved mitochondrial physiology and efficiency. We determined the status of mitochondrial membrane potential in *Indy^206^/+* and *Indy^206^/Indy^206^* mutant midgut tissue by using positively charged JC-1, which fluoresces green as a monomer in cytoplasm but forms red aggregates upon entering the mitochondrial matrix as a result of high membrane potential. The red:green ratio in aged *Indy* mutant midguts is significantly greater in both *Indy^206^/+* and *Indy^206^/Indy^206^* mutants compared to controls (Fig 2A,B). Moreover, the ratio for *Indy^206^/dPGC-1^KG08646^* double mutants is strikingly similar to that observed in controls suggesting that *dPGC-1* mediates changes in mitochondrial physiology observed in *Indy* mutants (Fig. 2A,B).

Increased levels of mitochondrial ROS production and oxidative damage are associated with decreased mitochondrial function and considered hallmarks of aging across species [2, 4, 23, 24]. Dihydroethidium (DHE) fluoresces red when it reacts with superoxide and was used to measure changes in the total redox environment in the midgut of female *Indy^206^* mutants, *Indy^206^/dPGC-1^KG08646^* double mutants and control flies at 40 days. Both *Indy^206^/+* and *Indy^206^/Indy^206^* mutants had significantly decreased red fluorescence when compared to controls indicating lower levels of ROS (Fig. 2C,D). *Indy^206^/dPGC-1^KG08646^* show partial protection against ROS accumulation as shown by intermediate levels of DHE intensity, which is likely due to the effects of *Indy* reduction on the wild-type copy of *dPGC-1* (Fig. 2D).

### *Indy* mutant flies have increased resistance to oxidative stress

We next examined whether *Indy* mutations affect fly oxidative stress resistance. ROS-detoxification factors *Glutathione S transferase E1* (*GstE1*) and *Glutathione S transferase D5* (*GstD5*) mRNA were significantly increased in *Indy^206^/+* and *Indy^206^/Indy^206^* mutant midgut tissue at 40 days compared to *yw* controls (Fig. 2E). Levels were not significantly altered in *Indy^206^/dPGC-1^KG08646^* flies compared to controls, suggesting that both copies of wild type *dPGC-1* are necessary to modulate ROS detoxification in *Indy* mutants (Fig. 2E). Additionally, male and female *Indy^206^* mutant flies have increased oxidative stress resistance when exposed to 20 mM paraquat compared to *yw* controls at 20 days (Fig. 2F, 2G, Table S1). Increased resistance in *Indy^206^/+* flies compared to *Indy^206^/Indy^206^* flies can be atributed to enhanced mitochondrial physiology and decreased ROS levels found in *Indy^206^*/+(Fig. 2A-2E).

### *Indy* mutations preserve ISC homeostasis and intestinal integrity

Robust INDY expression in the midgut suggests that transporting metabolites across the midgut epithelia is one of the main functions of INDY. The midgut is maintained by multipotent ISCs, which divide asymmetrically giving rise to an identical daughter ISC and an immature progenitor enteroblast cell (EB) with differentiation potential. Elevated levels of ROS disrupt ISC proliferation patterns by inducing division at a rate that surpasses EB differentiation, which leads to accumulation of polyploid aggregates, decreased intestinal integrity and accelerated mortality [5, 17, 18, 25]. The effect of decreased *Indy* expression on intestinal homeostasis was examined using the *Indy^YC0030^* mutant fly line, which has a fluorescent protein (GFP) tag inserted in the *Indy* gene region and was backcrossed 10 generations into the *yw* background [26]. *Indy^YC0030^/+* mutant flies have reduced *Indy* transcript in the midgut at levels similar to those observed in *Indy^206^/+* mutants. These flies also demonstrate significant longevity extension, with a 58.3% and 42.2% increases in median life span in males and females, respectively (Fig. S3A-D, Table S2).

**Figure 3:**
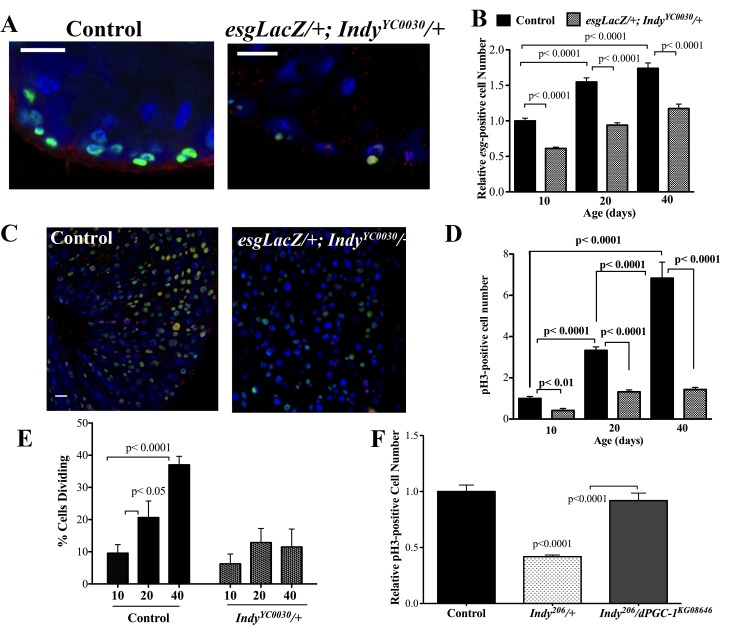
*Indy* mutations preserve ISC homeostasis. (**A**) Immunostaining for INDY (red) DAPI (blue nuclear) and β-galactosidase (green) in the midgut of female control (*esgLacZ/+*) and *Indy* mutant (*esgLacZ;Indy^YC0030^*/+) flies at 20 days at 40X. Scale bar represents 1μm. (**B**) Quantification shows reduced number of *esg*-positive cells in the *esgLacZ; Indy^YC0030^*/+ mutant female midgut throughout lifespan (p<0.0001, n>20). (**C**) Immunostaining for β-galactosidase activity (green), nuclear (blue) and pH3-positive cells (red) in control (*esgLacZ/+)* and the *Indy* mutant (*esgLacZ/+;Indy^YC0030^/+)* midgut tissue at 40 days. β-gal- positive cells represent ISC/EB populations and pH3-positive cells represent dividing cells. Scale bar represents 1μm. (**D**) Quantification of pH3-positive cells in the midgut of control (*esgLacZ/+)* and *Indy* (*esgLacZ;Indy^YC0030^*/+*)* mutant flies. There is increased cell division in female control midgut tissue throughout lifespan that is largely absent in *esgLacZ; Indy^YC0030^*/+ mutant females (p<0.01, p<0.0001, n>20). Error bars represent SEM. (**E**) Quantification of dividing cells in the midgut determined by the presence of pH3-positive immunostaining. There is increased cell division in female control midgut tissue throughout lifespan that is largely absent in *esgLacZ; Indy^YC0030^*/+ mutant females (p<0.05, p<0.0001, n>20). Error bars represent SEM. See Figures S4, S5 and Table S2. (**F**) Quantification of pH3*-* postive cells in the midgut of *yw* control, *Indy^206^/+* mutant and *Indy^206/+^/dPGC-1^KG08646^* female flies at 40 days. There are reduced dividing cells in the midgut of *Indy* mutant flies compared to control and *Indy^206^/dPGC-1^KG08646^* midgut (p<0.0001, n>15). Error bars represent SEM.

Both the ISC and the progenitor EB express the transcription factor *escargot (esg);* therefore *esg-* positive cells represent undifferentiated cells [20, 21]. The number of undifferentiated cells in the midgut was assessed using *esgLacZ* flies, which express βgal under an *esg* reporter and were backcrossed 10 generations into the *yw* genetic background. Quantification of βgal-positive cells in aging male and female control *esgLacZ/+* and *esgLacZ*;*Indy^YC0030^/+* mutant midgut tissue show significantly fewer βgal-positive cells in *Indy^YC0030^/+* flies compared to control flies at 40 days (Fig. 3A,B, S4A,B).

Phosphorylation of histone 3 (pH3) occurs during mitosis and marks active cell proliferation [27]. *esgLacZ;Indy^YC0030^/+* mutant flies have significantly fewer pH3-positive cells in midgut tissue compared to control *esgLacZ/+* flies at 40 days (Fig. 3C,D, S4B). Moreover, the number of pH3-positive cells is steady from 20-40 days in *Indy* mutant flies, indicating preserved ISC proliferative homeostasis (Fig. 3E). Using double mutant *Indy^206^*/*dPGC-1^KG08646^* flies, we determined that two copies of *dPGC-1* are required to maintain low numbers of pH3-positive cells in aged *Indy^206^* mutants. Unlike *Indy^206^/+* mutants, double mutant *Indy^206^*/*dPGC-1^KG08646^* midguts have significantly more pH3-positive cells, suggesting *dPGC-1* mediates downstream effect of *Indy* reduction (Fig. 3F).

**Figure 4:**
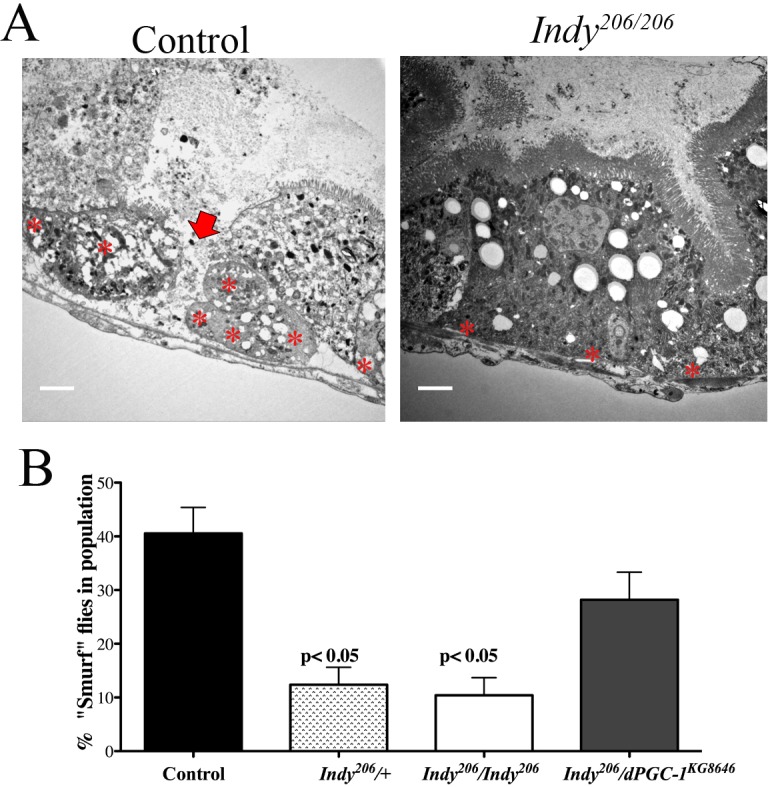
*Indy* mutations preserve intestinal integrity. (**A**) Electron micrograph of *yw* control (left) and *Indy^206^/Indy^206^* (right) midgut imaged at 1000x. Arrow shows damaged tissue and asterisks mark ISCs. Scale bar represents 1 μm. (**B**) Quantitative analysis of blue staining of female control and *Indy^206^/Indy^206^* flies fed food containing 2.5% w/v FD&C blue dye for 150 minutes at 40 days. Blue coloring throughout body indicates loss of intestinal integrity. Control and *Indy^206^*/*dPGC-1^KG08646^* flies have a significantly higher number of blue flies compared to *Indy^206^/+* and *Indy^206^/Indy^206^* mutants (p<0.01, n>50).

ISCs maintain intestinal architecture by replacing damaged cells that comprise the barrier between lumen and hemolymph in the midgut. Age-related accumulation of aggregates and regions of disjoined cells compromises intestinal integrity and can be observed in the midgut of control flies; however, *Indy^206^*/*Indy^206^* midguts retain cellular architecture (Fig. 4A,B). To assess the state of intestinal integrity in aged *Indy* mutants, we added 2.5% w/v non-absorbable FD&C blue dye #1 to fly food and quantified the percentage of flies displaying total tissue staining [17]. About 40% of aged controls show loss of intestinal integrity as measured by total staining throughout the body after feeding (Fig. 4C). In contrast, only about 10% of *Indy^206^*/*+* and *Indy^206^*/*Indy^206^* mutant flies were completely blue, with most retaining blue dye in the digestive tract and proboscis similarly to young flies (Fig. 4C). Furthermore, we observed about 30% of *Indy^206^/dPGC-1^KG08646^* flies with total blue staining, which further supports a role for *dPGC-1* as a downstream mediator of beneficial effects of *Indy* mutations.

### *Indy*-longevity is mediated by *dPGC-1*

**Figure 5:**
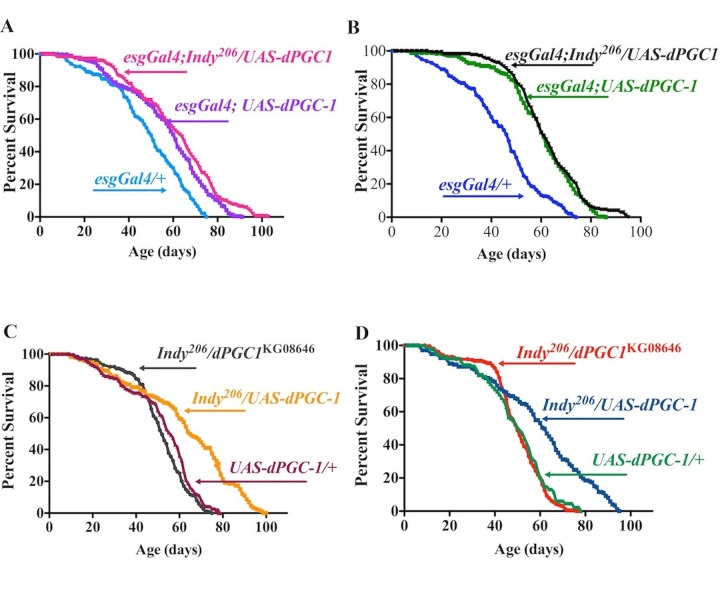
*Indy* and *dPGC-1* longevity pathways overlap. (**A**) Lifespan curves of female *esgGal4;Indy^206^/UAS-dPGC1* (magenta), *esgGal4;UAS-dPGC-1* (purple) and genetic controls (*esgGal4/+*) (blue) flies. *esgGal4;UAS-dPGC-1* females overexpressing *dPGC-1* in *esg*-positive cells, and *Indy* mutant females with *dPGC-1* overexpression in the *esg*-positive cells have 19.9%, and 27.7% increase in median longevity compared to genetic controls (*esgGal4/+*) flies, respectively. (**B**) Lifespan curves of male *esgGal4;Indy^206^/UAS-dPGC1* (black), *esgGal4;UAS-dPGC-1* (green) and genetic controls (*esgGal4/+*) (blue). *esgGal4;UAS-dPGC-1* males overexpressing *dPGC-1* in *esg*-positive cells, and *Indy* mutant male with *dPGC-1* overexpression in the *esg*-positive cells have 35.1% and 40.6% increase in median longevity compared to genetic controls (*esgGal4/+*), respectively. (**C**) Lifespan curves of female *Indy^206^* mutants with a hypomorphic allele for *dPGC-1* (*esgGal4;Indy^206^/UAS-dPGC1*) (gray), *Indy* mutant flies with one copy of the *dPGC-1UAS* construct (*esgGal4;Indy^206^/UAS-dPGC1*) (yellow) and genetic controls (*UAS-dPGC1/+*) (maroon). *esgGal4;Indy^206^/UAS-dPGC1* flies show similar longevity compared to controls and *esgGal4;Indy^206^/UAS-dPGC1* females show 22.5% median longevity extension. (**D**) Lifespan curves of male *Indy^206^* mutant with a hypomorphic allele for dPGC-1 (*esgGal4;Indy^206^/UAS-dPGC1*) (red), *Indy* mutant flies with one copy of the *dPGC-1UAS* construct (*esgGal4;Indy^206^/UAS-dPGC1*) (blue) and genetic controls (*UAS-dPGC1/+*) (green). *esgGal4;Indy^206^/UAS-dPGC1* flies show similar longevity compared to controls and *esgGal4;Indy^206^/UAS-dPGC1* males show 23.5% median longevity extension. See Figure S6, Table 3. n>170 for all lifespan studies.

Decreased *Indy* expression and restricted upregulation of *dPGC-1* in midgut stem and progenitor cells extends lifespan in flies [12, 13, 17]. As described above, *Indy* mutants have significantly increased *dPGC-1* mRNA levels in the midgut throughout lifespan (Fig. 1C); therefore we investigated whether these two longevity pathways share a similar mechanism. We used the *esgGAL4/UASdPGC-1*(*Spargel^EY05931^)* system to overexpress *dPGC-1* in stem and progenitor cells of the digestive tract [17, 28]. To avoid any effects of genetic background, all flies were backcrossed to *yw* background for 10 generations. Although *esgGal4* is also expressed in stem cells of malpighian tubules, the testis and in salivary glands, we focus our attention on the effects of overexpressing *dPGC-1* on midgut physiology due to its importance in healhy aging [25]. We censored the first 9 days following eclosion to reduce the influence of early, non-age associated death on longevity studies. Female and male *esgGal4;UAS-dPGC1* flies have increased median lifespan by 19.9% and 35.1%, respectively, compared to control *esgGal4/yw* flies (Fig. 5A,B, Table 3). If *Indy* and *dPGC-1* longevity pathways overlap, we would not expect that overexpression of *dPGC-1* in *esg*-positive cells of *Indy^206^* mutant flies (*esgGal4;Indy^206^/UAS-dPGC-1)* to further extend longevity of *Indy* mutant flies. Compared to controls, these flies have median lifespan extension of 27.7% and 40.6%, in females and males, respectively. As predicted, they do not experience additional increase in lifespan compared to *esgGal;UAS-dPGC-1* (Fig. 5A,B, S5A, Table 3).

Flies heterozygous for the *Indy^206^* allele with one copy of the *UAS-dPGC-1* construct (*Indy^206^/UAS-dPGC-1*) have median lifespan extension compared to the survivorship of *UAS-dPGC-1/yw* flies with 22.5% and 23.5% increases in female and male flies respectively (Fig. 5C, D, Table 3). Furthermore longevity extension was not observed in double mutant *Indy^206^/dPGC-1^KG08646^* flies compared to genetic control (*UAS-dPGC-1/yw*) and was signifcantly shorter compared to *Indy^206^/UAS-dPGC-1*, suggesting that the longevity extension observed in *Indy^206^* mutant flies with one copy of *Indy^206^* chromosome, is most likely mediated by increased levels of *dPGC-1* (Fig. 5C, D, Table 3). This conclusion is supported by findings that *dPGC-1* mRNA levels found in the midgut of *Indy* mutant flies are the same as those found in flies overexpressing *dPGC-1* in *esg*-positive cells in *Indy* mutants or flies with wild type INDY (Fig. S5). Together, the data support a model for *Indy*-mediated longevity that is mediated by downstream activation of *dPGC-1* and its effect on mitochondrial physiology.

## DISCUSSION

Reduction of *Indy* gene activity in fruit flies, and homologs in worms, extends lifespan by altering energy metabolism in a manner similar to CR [6-8, 11-14]. *Indy* mutant flies on regular food share many characteristics with CR flies and do not have further longevity extension when aged on a CR diet [14, 29, 30]. Furthermore, *mINDY^−/−^* mice on regular chow share 80% of the transcriptional changes observed in CR mice, supporting a conserved role for INDY in metabolic regulation that mimics CR and promotes healthy aging [7]. In this study we shifted from systemic to the tissue specific effects of INDY reduction, focusing on the midgut due to the high levels of INDY protein expression in wild type flies and the importance of regulated intestinal homeostasis during aging. Our evidence supports a role for INDY as a physiological regulator that senses changes in nutrient availability and alters mitochondrial physiology to sustain tissue-specific energetic requirements.

**Figure 6:**
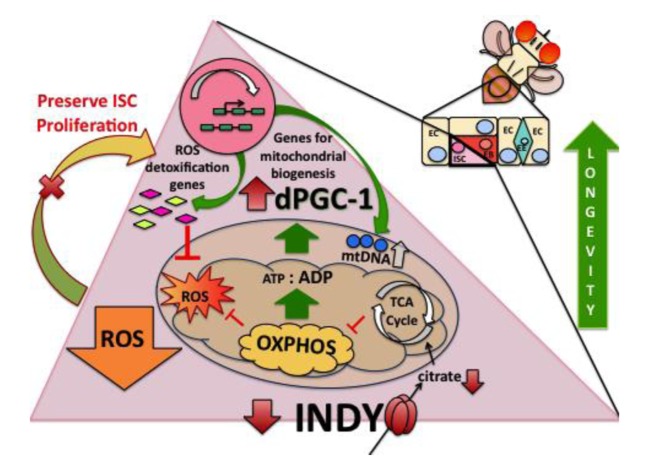
*Indy* mutations preserve ISC homeostasis. INDY transports citrate from hemolymph into the cells and vice versa. Cytoplasmic citrate can be transported to mitochondria and used as a substrate for the TCA cycle. Reduced INDY-mediated transport decreases citrate levels and decreases the ATP/ADP ratio. Such changes activate AMPK, promoting fat oxidation and *dPGC-1* synthesis. The increase in *dPGC-1* activity increases mitochondrial biogenesis and transcription of ROS-detoxification genes. Decreased ROS production preserves ISC homeostasis, which contributes to *Indy* mediated longevity extension.

We show an age-associated increase in midgut *Indy* mRNA levels that can be replicated by manipulations that accelerate aging such as increasing the caloric content of food or exposing flies to paraquat. Conversely, we show that CR decreases *Indy* mRNA in control midgut tissues, which is consistent with previous findings in fly muscle and mouse liver [7, 14]. Diet-induced variation in midgut *Indy* expression suggests that INDY regulates intermediary metabolism by modifying citrate transport to meet tissue or cell-specific bioenergetic needs. Specifically, as a plasma membrane transporter INDY can regulate cytoplasmic citrate, thereby affecting fat metabolism, respiration, and via conversion to malate, the TCA cycle. Recent reports show that pluripotent stem cells use intermediate metabolites, from the TCA cycle, such as citrate, to propagate proliferation [31, 32]. Reduced INDY-mediated transport activity in the midgut could prevent age-related ISC-hyperproliferation by decreasing the available energy needed to initiate proliferation, thereby preserving tissue function during aging (Fig. 6). This is supported by findings that nutrient availability affects ISC proliferation in adult flies and that CR can affect stem cell quiescence and activation [19, 33].

One of the hallmarks of CR-mediated longevity extension is increased mitochondrial biogenesis mediated by *dPGC-1* [1-3]. Increased *dPGC-1* levels and mitochondrial biogenesis have been described in the muscle of *Indy* mutant flies [6], the liver of *mIndy^−/−^* mice [7], and here we describe it in the midgut of *Indy* mutant flies. One possible mechanism for these effects can be attributed to the physiological effects of reduced INDY transport activity. Reduced INDY-mediated transport activity could lead to reduced mitochondrial substrates, an increase in the ADP/ATP ratio, activation of AMPK, and *dPGC-1* synthesis. This is consistent with findings in CR flies and the livers of *mINDY^−/−^* mice. Our analysis of mitochondrial physiology in the *Indy* mutant midgut shows upregulation of respiratory proteins, maintenance of mitochondrial potential and increased mitochondrial biogenesis, all of which are signs of enhanced mitochondrial health [4]. The observed increase in *dPGC-1* levels in *Indy* mutant midgut therefore appears to promote mitochondrial biogenesis and functional efficiency, representing a protective mechanism activated in response to reduced energy availability.

Genetic interventions that conserve mitochondrial energetic capacity have been shown to maintain a favorable redox state and regenerative tissue homeostasis [17, 18, 34, 35]. This is particularly beneficial in the fly midgut, which facilitates nutrient uptake, waste removal and response to bacterial infection. *Indy* mutant flies have striking increases in the steady-state expression of the *GstE1* and *GstD5* ROS detoxification genes. As a result, any increase in ROS levels, whether from mitochondrial demise or exposure to external ROS sources can be readily metabolized to prevent accumulation of oxidative damage. Such conditions not only promote oxidative stress resistance, but also preserve ISC homeostasis as demonstrated by consistent proliferation rates throughout *Indy* mutant lifespan and preserved intestinal architecture in aged *Indy* mutant midguts. Thus, enhanced ROS detoxification mechanisms induced by *Indy* reduction and subsequent elevation of *dPGC-1* contributes to preservation of ISC functional efficiency, and may be a contributing factor to the long-lived phenotype of *Indy* mutant flies.

Several lines of evidence indicate that INDY and *dPGC-1* are part of the same regulatory network in the midgut, in which *dPGC-1* functions as a downstream effector of INDY. The similarity between *dPGC-1* mRNA levels and survivorship of flies overexpressing *dPGC-1* in *esg*-positive cells and *Indy* mutant flies suggests that *Indy* and *dPGC-1* interact to extend lifespan. This is further supported by the lack of additional longevity extension when *dPGC-1* is overexpressed in *esg*-positive cells of *Indy* mutant flies. Moreover, hypomorphic *dPGC-1* flies in an *Indy* mutant background are similar to controls with respect to life span, declines in mitochondrial activity and ROS-detoxification. Together, these data suggest that *dPGC-1* must be present to mediate the downstream physiological benefits and lifespan extension of *Indy* mutant flies.

There are some physiological differences between the effects of *Indy* mutation and *dPGC-1* overexpression in *esg-*positive cells [17]. While *Indy* mutant flies are less resistant to starvation and more resistant to paraquat, a recent report showed that overexpressing *dPGC-1* in *esg-*positive cells has no effect on resistance to starvation or oxidative stress [14, 17]. Additionally, mice lacking skeletal muscle PGC-1α were found to lack mitochondrial changes associated with CR but still showed other CR-mediated metabolic changes [36]. In the fly INDY is predominantly expressed in the midgut, fat body and oenocytes, though there is also low level expression in the malpighian tubules, salivary glands, antenae, heart and female follicle cell membranes. Thus, the effects of INDY on intermediary metabolism and longevity could be partially independent from *dPGC-1* or related to changes in tissues other than the midgut.

Our studies suggest that INDY may function as a physiological regulator of mitochondrial function and related metabolic pathways, by modulating nutrient flux in response to nutrient availability and energetic demands. Given the localization of INDY in metabolic tissues, and importance of regulated tissue homeostasis during aging, these studies highlight INDY as a potential target to improved health and longevity. Reduced *Indy* expression causes similar physiological changes in flies, worms and mice indicating its regulatory role would be conserved. Further work should examine the interplay between *Indy* mutation and metabolic pathways, such as insulin signaling, which have been shown to promote stem cell maintenance and healthy aging in flies and mice [25, 35, 37]. In doing so, the molecular mechanisms, which underlie *Indy* mutant longevity may provide insight for anti-aging therapies.

## METHODS

### Fly Strains

The *Indy^YC0030^* line was obtained from Lynn Cooley [26]. The *esgGal4 (y^1^w;esgGAL4/Cyo)(#26816), esgLacZ (y^1^w^67c23^;esgLacZ/Cyo) (#10359), UAS-dPGC-1 (yw; Spargel ^EY05931^) (#2009*), *y^1^*;*P{SUPor-P}Spargel^KG08646^ry^506^/TM3,Sb^1^Ser^1^* *(#14965)* and *yellow-white (yw)* flies were obtained from the Bloomington Stock Center at Indiana University. The *Indy^206^* line was obtained from Tim Tully [38]. The *TIGS-2* Gene-Switch driver line was provided by Scott Pletcher and the UAS-*Indy^RNAI^* (*w^1118^;P{GD2712}v9981)* line was obtained from the Vienna *Drosophila* RNAi Center [39].

### Fly Maintenance and Lifespan Studies

Flies were collected within 24 hours after eclosion and maintained in plastic vials containing standard food medium and kept in a humidified, temperature-controlled incubator with 12/12-h on/off light cycle at 25 °C. All strains were backcrossed 10x to *yellow-white (yw)* background and reared on food containing 25 mg/mL tetracycline for 3 generations to eliminate *Wolbachia*, followed by several generations in tetracycline-free food. Lifespan studies were performed using 10 groups of 25 male and 25 female flies, which were collected within 24 hours following eclosion as described above and maintained in plastic vials containing standard, high or low calorie food medium and kept in a humidified, temperature-controlled incubator with 12/12-h on/off light cycle at 25 °C. Flies were transferred to fresh food every other day for the first 30 days and then every day until no flies remained alive. The number of dead flies was scored after each passage. Flies requiring gene-switch induction were grown on food containing 200 μM RU486 and controls on EtOH. Approximately 20 females and 20 males flies are kept together in each vial and passed to fresh vials every 2 days for aging studies.

Longevity data were censored for early mortality (1-9 Days) and analyzed by long-rank tests using the JMP 10 program.

### Oxidative Stress Resistance Studies

Oxidative stress resistance studies were conducted by keeping 20 flies in a vial containing filter paper soaked with 300 μL of 20 mM paraquat following initial starvation for 6 hours. The number of dead flies was counted hourly during the day and twice overnight until no flies remained alive. Stress resistance data were analyzed by long-rank tests using the JMP 10 program. Total number of flies per experiment is listed in Table 1.

### Quantitative PCR (qPCR)

Total RNA was isolated from the midguts of 3 biological replicates with more than 25 flies in each replicates using Trizol as described [14]. qPCR was performed following cDNA synthesis and changes in gene expression patterns were determined using a 7500 Fast Real-Time PCR System (Applied Biosystems) and TaqMan Master Mix (Applied Biosystems). Gene specific TaqMan primers for *Indy, dPGC-1, Pdsw, l(3)neo18, COI, ND23, ND42, ND75, GstD5 and GstD1* were obtained from the Invitrogen. All experiments were run in triplicate. *Ankryn* was used as an endogeneous controls in all q-PCR experiments.

### Mitochondrial DNA Measurement

Total DNA from the midguts of more than 25 flies was isolated at 40 days using the Invitrogen DNA blood and tissue isolation kit (Life technologies). DNA copy number was determined using qPCR as described above. Mitochondrial DNA content was determined by the ratio of the mitochondrial gene for *COI* to a nuclear gene, *GAPDH* [6]. *Rpl1140* was used as an endogenous control.

### Electron Microscopy and Mitochondrial Quantification

Flies were fixed in 2% glutaraldehyde in 0.1 M sodium cacodylate buffer as described [21]. A minimum of 15 electron micrographs of midgut sections of each sample were taken at 10,000-15,000x, using an unbiased sampling method. Images were processed and analyzed in Adobe Photoshop. Post-fixation was conducted for 1 hr in 1% osmium tetroxide-0.8% potassium ferricyanide. Samples were stained in block with 1% aqueous uranyl acetate, dehydrated in a graded ethanol series, and embedded in Spurr low-viscosity epoxy resin. Thin sections of areas containing midgut were stained with uranyl acetate and lead citrate, and examined in a Hitachi H7650. Mitochondria were counted by using the point counting method by using a grid system to count the number of mitochondria present in a given image relative to cytoplasmic volume [7]. Size was determined by measuring the grid overlays per mitochondria and expressed relative to cytoplasmic volume.

### Immunostaining, Quantification of ISCs/EBs and pH3+ Cells

Midguts were dissected from flies at 10, 20 and 40 days, fixed in 4% paraformaldehyde and stained as described [17]. Following washing, samples were mounted and imaged using the Leica camera attachment using LAS V4.1 software, or the Zeiss 780 combined confocal/FCS/NLO system, mounted on an inverted Axio Observer Z1. Fixed tissue was incubated overnight with mouse anti-gal 1:500 (Invitrogen); rabbit anti-pH3 1:300 (Invitrogen) or rabbit anti-INDY 1:300 [10] primary antibodies diluted in PBT [0.1%Triton X-100 in phosphate-buffered saline (PBS)] at 4°C. Following washing and blocking, tissue was incubated with the goat anti-rabbit Cy3 1:300 (Jackson) or goat anti-mouse FITC 1:300 (Jackson) secondary antibodies and DAPI 1:1000 (Invitrogen) diluted in PBT and 2% donkey serum for 1 hour at room temperature. Images were analyzed using Adobe Photoshop or Image J. Variability between different regions of the gut was reduced by quantifying images from the same designated region for each genotype in a 0.06x 0.02cm area. Cells were counted, values averaged and standard deviation calculated separately.

### Dihydroethidium Staining

ROS levels were assessed in live whole midgut tissue as described [17, 18]. Tissue was dissected directly in Schneider's medium and incubated for 7 minutes in 60 mM dihydroethidium (DHE) (Invitrogen Molecular Probes) in Schneider's medium and 1:1000 4',6-diamidino-2-phenylindole (DAPI) nuclear stain in 0.1% PBT 2% donkey serum. Midguts were washed in Schneider's medium at room temperature, mounted in 70% glycerol and imaged using a Zeiss 780 combined confocal/FCS/NLO system, mounted on an inverted Axio Observer Z1. 2 μm Z stacks of regions 200-500 μm anterior to the pylorus were measured for mean signal intensity at 568 nm in Image J. Pixel intensities of Z stacks, spanning from the basal to apical cell layers, for at least 15 midguts per genotype were used for each of the quantifications.

### JC-1 Analysis

JC-1 analysis was performed as described [17]. Whole midguts were dissected from female flies at 40 days directly into 5 μM JC-1 (Molecular Probes) in DMSO containing 1:1000 DAPI (Invitrogen) and incubated in the dark for 30 minutes at room temperature. Midguts were washed 2 times for 5 minutes each and mounted in PBS. Images were taken of midguts approximately 300 μm from the anterior pylorus in the 568 nm channel using the Zeiss 780 combined confocal/FCS/NLO system and analyzed in Image J. Mean pixel intensities for J-aggregates were averaged, and significant differences between means were determined with a Mann-Whitney U test.

### Intestinal Integrity

Quantification of intestinal integrity was done as described [17]. More than 50 female flies were transferred to standard lab food containing 2.5% w/v FD&C blue dye #1 for 150 minutes beginning at 7am. The percentage of blue flies per population was quantified and represented as mean averages ± SE values.

### Statistical analysis

Significance was determined using a two-tailed, unpaired t-test from at least three independent experiments and expressed as P values, with the exception of longevity studies and JC-1 aggregation analysis. Error bars represent SEM, t test, P values are specifically indicated in each figure.

## SUPPLEMENTAL FIGURES AND TABLES


