# Supplementary material for: Increased mitochondrial biogenesis preserves intestinal stem cell homeostasis and contributes to longevity in Indy mutant flies
Source: Aging (Albany NY). 2014 May 6;6(4):335–50. doi: 10.18632/aging.100658 (PMC4032799; doi:10.18632/aging.100658)
Supplement: Supplementary file 1 [file aging-06-335-s001.pdf]

SUPPLEMENTARY FIGURES AND TABLES

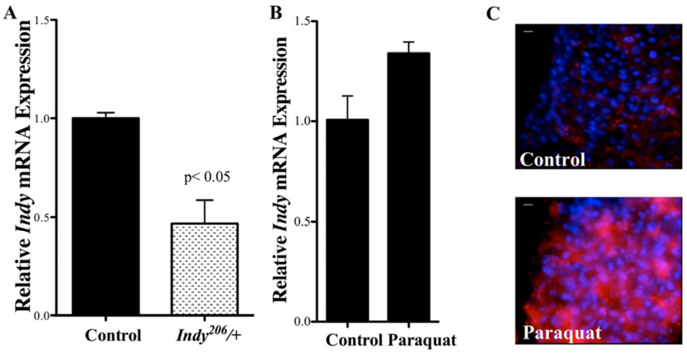

Figure S1. There is an age-related increase in *Indy* mRNA levels. (**A**) *Indy* mRNA levels in the midgut of male heterozygous *Indy206/+* mutant flies measured by qPCR at 40 days. There is significant reduction of *Indy* mRNA in the male mutant midgut (p<0.05, n=3, 25 guts per replicate). (**B**) *Indy* mRNA levels in the midgut of *yw* control male flies following overnight exposure to paraqaut at 20 days determined by qPCR. There is a paraquat-induced increase in *Indy* mRNA levels in the midgut of control flies (n=3, 25 guts per replicate). (**C**) Immunofluorescence images of a control female (top) and a female following exposure to 20mM (bottom) at 20 days, viewed with 40x oil immersion objective and stained with anti-INDY antibodies. INDY is increased following exposure to paraquat in the midgut.

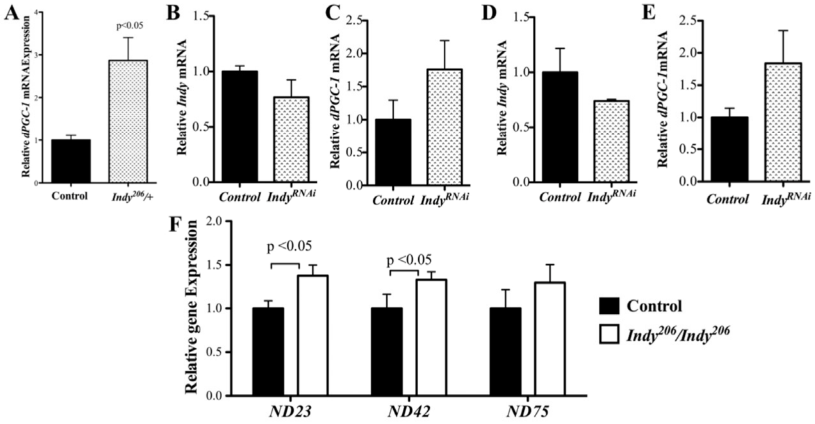

Figure S2. *dPGC-1* mRNA levels increase when *Indy* mRNA levels are reduced. (**A**) *dPGC-1* mRNA levels in the midgut of heterozygous *Indy206/+* males at 40 days. There is a significant increase in *dPGC-1* mRNA levels in *Indy206/+* male midgut tissue (p<0.05, n=3, 25 guts per replicate). (**B**) *Indy* mRNA levels in the midgut of female *TIGS2- GS*;*Indy9981* RNAi lines at 20 days measured by qPCR. (**C**) *dPGC-1* mRNA levels in the midgut of female *TIGS2-GS*;*Indy9981* RNAi lines at 20 days measured by qPCR. (**D**) *Indy* mRNA levels in the midgut of male *TIGS2-GS*;*Indy9981* RNAi lines at 20 days measured by qPCR. (**E**) *dPGC-1* mRNA levels in the midgut of male *TIGS2-GS*;*Indy9981* RNAi lines at 20 days measured by qPCR. *IndyRNAi* flies show a small increase in *dPGC-1* mRNA compared to control flies suggesting that targeted depletion of *Indy* in the midgut increases *dPGC-1* transcription (n=3, 25 guts per replicate). Error Bars represent SEM. (**F**) Quantification of Complex I genes *ND23, ND42* and *ND75* mRNA in the midgut of 2 control and *Indy206/Indy206* mutant flies determined by qPCR. *ND23* and *ND42* mRNA are significantly increased in the *Indy* mutant midgut at 20 days (p<0.05, n=3, 25 guts per replicate).

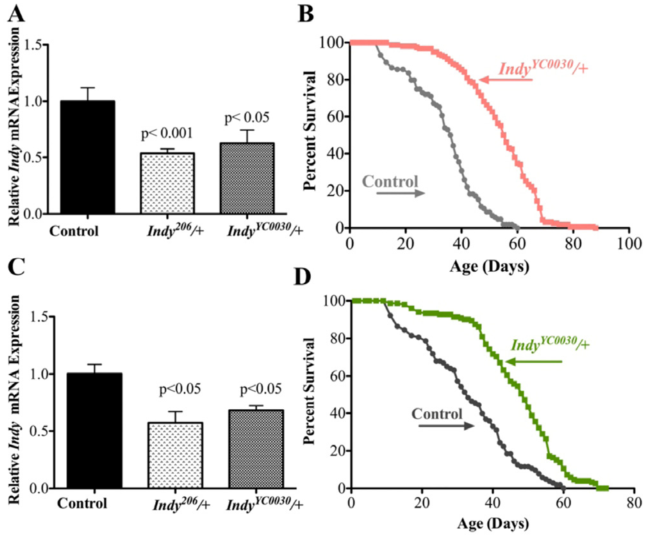

Figure S3. Characterization of *IndyYC0030/+* heterozygous mutant flies. (**A**) *Indy* mRNA levels in the midgut of *yw* control, *Indy206/+* and *IndyYC0030/+*female flies aged 20 days determined by qPCR. There is a significant (p<0.001, p<0.05, n=3, 25 guts per replicate) decrease in *Indy* mRNA levels in the midgut of *Indy206/+* and *IndyYC0030/+.* Error Bars represent SEM. (**B**) Life-span curves of control (gray) and *IndyYC0030/+* females (pink). A 58.3% increase in median survival was observed in *IndyYC0030/+* females. (**C**) Endogenous *Indy* mRNA levels in the midgut of control, *Indy206/+* and *IndyYC0030/+* male flies aged 20 days determined by qPCR. There is a significant (p<0.05, n=3, 25 guts per replicate) decrease in *Indy* mRNA levels in the midgut of *Indy206/+* and *IndyYC0030/+*. Error Bars represent SEM. (**D**) Life-span curves of control (gray) and *IndyYC0030/+*males (green). A 46.8% increase in median survival was observed in *IndyYC0030/+* males.

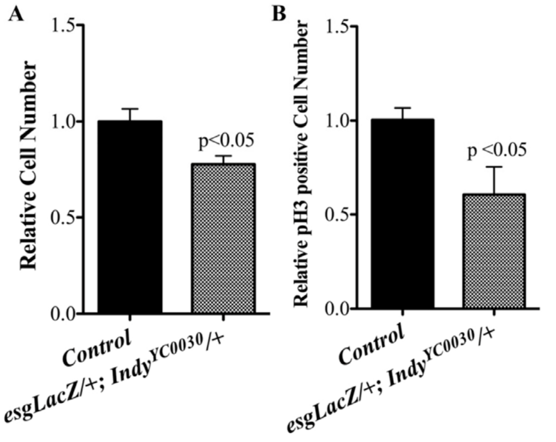

SFigure 4

Figure S4. Reduced INDY modulates intestinal homeostasis. (**A**) Quantification of *esg*positive cells in the midgut of control (*esgLacZ/+*) and *Indy* mutant (*esgLacZ;IndyYC0030/+*) male flies at 40 days. There are reduced ISC/EBs in the midgut of *Indy* mutant flies (p<0.05, n>15). (**B**) Quantification of pH3-postive cells in the midgut of control (*esgLacZ/+*) and *Indy* mutant (*esgLacZ;IndyYC0030/+*) male flies at 40 days. There are reduced dividing cells in the midgut of *Indy* mutant flies (p<0.05, n>15). Error bars represent SEM.

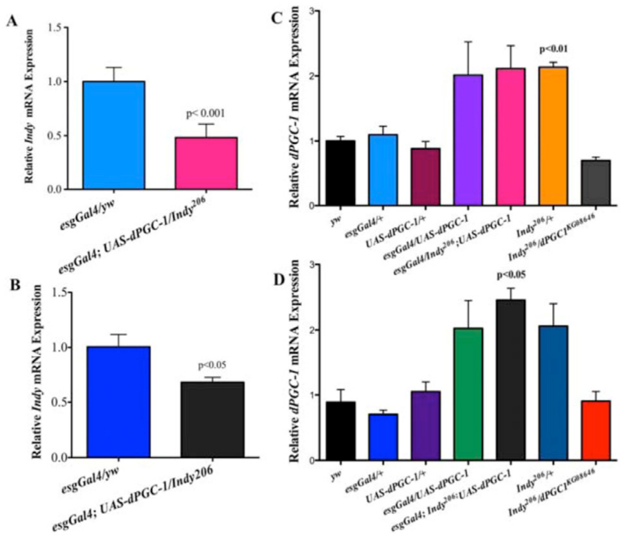

Figure S5. *Indy* and *dPGC-1* longevity pathways overlap. (**A**) Relative *Indy* mRNA levels measured by qPCR. *Indy* is significantly decreased in *esgGal4;Indy206/UAS-dPGC- 1* females and (**B**) males *at* 20 days compared to control (p<0.001, p<0.05, n=3, 25 guts per replicate). (**C**) Relative *dPGC-1* mRNA levels in the midguts of females (**C**) and males (**D**) measured by qPCR. *dPGC-1* is significantly increased in *esgGal4;UAS-dPGC-1, Indy206;UAS-dPGC-1 and esgGal4;Indy206/UAS-dPGC-1* and significantly decreased in *Indy206/dPGC-1KG08646,* compared to control *esgGal4/+* and UAS-dPGC-1/+ flies (p<0.05, p<0.001 n=3, 25 guts per replicate) but not between groups. Error bars represent SEM.

Table S1: *Indy* mutant flies are more resistant to paraquat stress.

Gender	Genotype	N	Median Lifespan (% Change)	X^2	*p*	Maximal Lifespan (% Change)
**F**	*yw*	95	15.1	-	-	21
**F**	$Indy^{206}/+$	85	19.8 (31.3)	62.2	p< 0.0001	26.8 (27.6)
**F**	$Indy^{206}/Indy^{206}$	85	17.1 (13.2)	24.3	p< 0.0001	24.4 (16.1)
**M**	*yw*	80	13.5	-	-	19.9
**M**	$Indy^{206}/+$	80	19.4 (43.7)	25.9	p< 0.0001	24.4 (22.6)
**M**	$Indy^{206}/Indy^{206}$	70	16.1 (19.2)	1.81	p=0.1787	22.1 (11.05)

The median and maximal lifespan of female (F) and male (M) *yellow-white (yw)* control, heterozygous ($Indy^{206}/+$) and homozygous ($Indy^{206}/Indy^{206}$) *Indy* mutant flies in *yw* background. N: number of flies in each lifespan. Median and Maximal Lifespans are in hours. Long-rank analyses were performed using the JMP 10 program.

Table S2. Heterozygous $Indy^{YC0030}/+$ mutant flies have increased longevity compared to *yw* genetic control flies.

Gender	Genotype	N	Median Lifespan (% Change)	X^2	*p*	Maximal Lifespan (% Change)
**F**	*yw*	160	34.1	-	-	53.5
**F**	$Indy^{YC0030}/+$	180	54 (58.3)	148.5	p< 0.0001	72 (34.5)
**M**	*yw*	145	32.9	-	-	55.6
**M**	$Indy^{YC0030}/+$	160	46.8 (42.2)	61.2	p<0.0001	64.8 (16.4)

The median and maximal lifespan of female (F) and male (M) *yellow-white (yw)* control and heterozygous *Indy* ($Indy^{YC0030}/+$) mutant flies in *yw* background. N: number of flies in each lifespan. Median and maximal lifespans are in days. Long-rank analyses were performed using the JMP 10 program.

Table S4: ***dPGC-1*** **is required for** ***Indy*** **mutant longevity extension**

Gender	Genotype	N	Median Lifespan (% Change)	X^2	*p*	Maximal Lifespan (% Change)
F	*UAS-dPGC-1/+*	150	51.5	-	-	73.3
F	$Indy^{206}$/*UAS-dPGC-1*	173	63.1 (22.5)	58.48	p< 0.001	95.9 (30.8)
F	$Indy^{206}$/*UAS-dPGC-*$1^{KG08646}$	188	51.8 (0.58)	5.19	p=0.022	70.1 (-4.3)
M	*UAS-dPGC-1/+*	145	48.5	-	-	71.3
M	$Indy^{206}$;*UAS-dPGC-1*	173	59.9 (23.5)	84.21	p<0.0001	92.9 (30.2)
M	$Indy^{206}$/*UAS-dPGC-*$1^{KG08646}$	205	49.2 (1.4)	1.21	p=0.27	66.7 (-6.5)

The median and maximal lifespan of female (F) and male (M) control *UAS-dPGC-1/+*: heterozygous flies with the *UAS-dPGC-1* construct in *yellow white* background. $Indy^{206}$/ *UAS-dPGC-1* flies have *UAS-dPGC-1* construct in $Indy^{206}$ mutant background. $Indy^{206}$/*UAS-dPGC-*$1^{KG08646}$ flies were generated by crossing $Indy^{206}$ flies to *dPGC-*$1^{KG08646}$ flies (hypomorph for the *dPGC-1).* All values are compared to either male or female *UAS-dPGC-1/+* control groups to determine the percent increase in median and maximal lifespan. *N*: number of flies used in the experiment. Median and maximal lifespan are in days. Data are censored for 0-9 days. Long-rank analyses were performed using the JMP 10 program.

Table S4: ***dPGC-1*** **is required for** ***Indy*** **mutant longevity extension**

Gender	Genotype	N	Median Lifespan (% Change)	X^2	*p*	Maximal Lifespan (% Change)
F	*UAS-dPGC-1/+*	150	51.5	-	-	73.3
F	$Indy^{206}$/*UAS-dPGC-1*	173	63.1 (22.5)	58.48	p< 0.001	95.9 (30.8)
F	$Indy^{206}$/*UAS-dPGC-*$1^{KG08646}$	188	51.8 (0.58)	5.19	p=0.022	70.1 (-4.3)
M	*UAS-dPGC-1/+*	145	48.5	-	-	71.3
M	$Indy^{206}$;*UAS-dPGC-1*	173	59.9 (23.5)	84.21	p<0.0001	92.9 (30.2)
M	$Indy^{206}$/*UAS-dPGC-*$1^{KG08646}$	205	49.2 (1.4)	1.21	p=0.27	66.7 (-6.5)

The median and maximal lifespan of female (F) and male (M) control *UAS-dPGC-1/+*: heterozygous flies with the *UAS-dPGC-1* construct in *yellow white* background. $Indy^{206}$/ *UAS-dPGC-1* flies have *UAS-dPGC-1* construct in $Indy^{206}$ mutant background. $Indy^{206}$/*UAS-dPGC-*$1^{KG08646}$ flies were generated by crossing $Indy^{206}$ flies to *dPGC-*$1^{KG08646}$ flies (hypomorph for the *dPGC-1).* All values are compared to either male or female *UAS-dPGC-1/+* control groups to determine the percent increase in median and maximal lifespan. *N*: number of flies used in the experiment. Median and maximal lifespan are in days. Data are censored for 0-9 days. Long-rank analyses were performed using the JMP 10 program.
